# Simultaneous Use of Cannulated Reamer and Schanz Screw for Closed Intramedullary Femoral Nailing

**DOI:** 10.5402/2011/502408

**Published:** 2011-04-11

**Authors:** Rajesh Rohilla, Roop Singh, Narender K. Magu, Ashish Devgan, Ramchander Siwach, Sukhbir Singh Sangwan

**Affiliations:** Department of Orthopaedic Surgery, Paraplegia & Rehabilitation, Pt. B.D. Sharma PGIMS, Haryana, Rohtak 124001, India

## Abstract

*Introduction*. Closed reduction is a critical component of the intramedullary nailing and at times can be difficult and technically challenging resulting in increased operative time. Fluoroscopy is used extensively to achieve closed reduction which increases the intra-operative radiation exposure. *Materials and Methods*. Sixty patients with femoral diaphyseal fractures treated by locked intramedullary nailing were randomized in two groups. In group I, fracture reduction was performed under fluoroscopy with a cannulated reamer in the proximal fragment or with simultaneous use of a cannulated reamer in the proximal fragment and a Schanz screw in the distal fragment. Patients in group II had fracture reduction under fluoroscopy alone. *Results*. Closed reduction was achieved in 29 patients in group I and 25 patients in group II. The guide wire insertion time, time for nail insertion and its distal locking, total operative time, and total fluoroscopic time were 26.57, 27.93, 68.03, and 0.19 minutes in group I, compared with 30.87, 27.83, 69.93, and 0.24 minutes in group II, respectively. The average number of images taken to achieve guide wire insertion, for nail insertion and its locking and for the complete procedure in group I, respectively, was 12.33, 25.27, and 37.6 compared with 22.1, 26.17, and 48.27, respectively, in group II. *Conclusion*. The use of cannulated reamer in proximal fragment as intramedullary joystick and Schanz screw and in the distal fragment as percutaneous joystick facilitates closed reduction of the fracture during closed intramedullary femoral nailing with statistically significant reduction in guide wire insertion time and radiation exposure.

## 1. Introduction

The femoral shaft fractures in adults are preferably treated with closed intramedullary nailing [1–4]. Closed reduction is a critical component of the procedure [5], and numerous techniques and devices have been proposed to aid closed reduction, in an attempt to overcome some of the associated problems [2, 3, 5–23]. Fracture table generates longitudinal traction to achieve closed reduction and maintains the reduction during the operative fixation [16, 21]. But use of a fracture table prolongs prep and drapes time, operative time, and anesthesia time [4]. Intramedullary nailing of femur without a fracture table has been reported [4, 7, 9, 11]. Compared with fracture-table traction, manual traction for intramedullary nailing of isolated fractures of the femoral shaft has been shown to decrease operative time and improve the quality of the reduction [9, 22]. Fluoroscopy is used extensively to achieve closed reduction and locking during locked intramedullary nailing which increases the intraoperative radiation exposure [24, 25]. Weil et al. recently reported that computerized navigation has the potential for increasing precision in fracture reduction while minimizing fluoroscopic requirements [5], but this facility is not available in every institution. A small diameter nail in the proximal fragment [3, 10, 14, 22] or 8 mm straight reamer into the proximal fragment [7] and a Schanz pin as percutaneous skeletal joystick in either of the fragments [2] have been used to assist closed reduction. However, simultaneous use of cannulated reamer in proximal fragment as intramedullary joystick and Schanz screw in the distal fragment as percutaneous joystick has never been reported earlier. Moreover, the quantitative impact of these techniques on success of closed reduction and reduction of radiation exposure has rarely been documented.

The purpose of this study was to compare prospectively the duration of nailing procedure: number of radiation exposures or images required for closed reduction in patients operated with a cannulated reamer in the proximal fragment or with simultaneous use of a cannulated reamer in the proximal fragment and a Schanz screw in the distal fragment versus those operated without it.

## 2. Materials and Methods

In a prospective study from March 2006 to December 2008, 60 patients (35 males and 25 females) with femoral diaphyseal fractures were operated with closed interlocked nailing after stratified randomization into two groups. All patients gave the informed consent prior being included into the study. The study was authorized by the local ethical committee and was performed in accordance with the Ethical standards of the 1964 Declaration of Helsinki as revised in 2000. After initial management in Accident and Emergency Department, all patients were put on skeletal traction through upper tibial Steinman pin with weights of 7 to 12 kg till operation. To maintain uniformity, we tried to perform all procedures under identical operative conditions. All patients were operated by same surgeons (first and second authors). As per AO classification 16, 20, and 24 fractures were type 32 A, B, and C, respectively. After fracture classification, the patients were randomized to one of the two treatment groups using lottery method. Group I contained 30 patients (17 males and 13 females) with average age of 44.6 years (range, 22–66 years). Group II contained 30 patients (18 males and 12 females) with average age of 41.2 years (range, 24–65 years). As per AO classification type A, fractures were 8 and 8; type B9 and 11; and 13 and 11 fractures were type C in groups I and II, respectively. Operations were performed on ordinary operation table under image intensifier control in lateral decubitus with the fractured leg uppermost. In group I, fracture reduction was performed under fluoroscopy with a cannulated reamer in the proximal fragment or with simultaneous use of a cannulated reamer in the proximal fragment and a Schanz screw in the distal fragment. Patients in group II had fracture reduction under fluoroscopy without using these devices.

### 2.1. Surgical Technique

**Figure 1:**
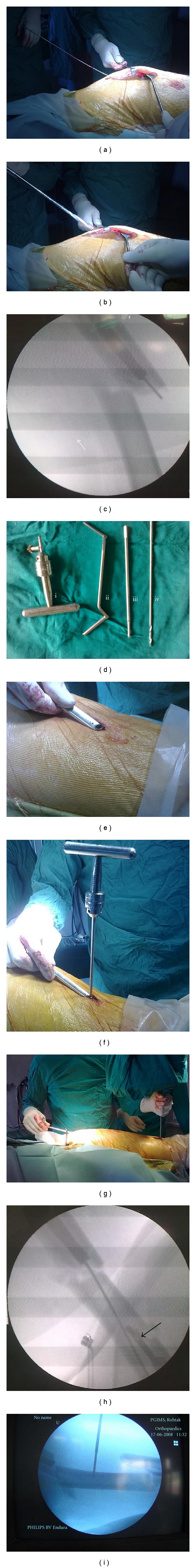
Surgical technique: (a) Insertion of the guide wire in the proximal fragment. (b) Insertion of the cannulated reamer over guide wire. (c) The cannulated reamer over the guide wire is used as intramedullary joystick to control the proximal fragment as visualized under image intensifier. (d) Instruments used to insert percutaneous Schanz screw to aid fracture reduction (i) T handle, (ii) Drill sleeve, (iii) 4.5 mm cortical Schanz screw (iv) 3.2 mm drill bit. (e) Insertion of the Drill sleeve up to the bone. (f) T handle with Schanz screw inserted in the lateral cortex of the distal fragment. (g) The surgeon controls the distal fragment with the Schanz screw and the proximal fragment with the help of cannulated reamer to achieve fracture reduction. (h) Insertion of the guide wire through the cannulated reamer into the distal fragment. The arrow depicts the Schanz screw in the distal fragment. (i) The Schanz screw is inserted in the intermediate fragment in segmental diaphyseal fractures (Type C2 fractures).

A 5–8 cm skin incision is made extending proximally from the greater trochanter. The tensor fascia lata and the abductor muscles are split along the incision down to the greater trochanter to expose the piriform fossa. The proximal femoral canal is entered through piriform fossa using a curved awl. 8 mm and 9 mm straight stiff handheld reamers are used to enlarge the proximal femoral canal. A guide wire is inserted into the proximal fragment after removal of stiff reamer (Figure 1(a)). A guide wire introducer or T-handle can be attached at proximal end of the guide wire to control its movements.

### 2.2. Techniques for Fracture Reduction and Insertion of the Guide Wire into the Distal Fragment under Image Intensifier Control

**Group I**

9 mm straight stiff handheld cannulated reamer is inserted over the guide wire in the proximal fragment (Figure 1(b)). Fracture is reduced with traction through skeletal pin. The cannulated reamer in the proximal fragment is used as intramedullary joystick to control the proximal fragment (Figure 1(c)). The insertion of the guide wire into the distal fragment is attempted up to a maximum of 7-8 images of the C-arm using the cannulated reamer as intramedullary joystick in the proximal fragment. In case of difficulty in guide wire insertion in the distal fragment, following maneuver is performed to assist fracture reduction. The instruments used in this maneuver are shown in Figure 1(d). A stab incision is given on the lateral aspect of distal fragment about 3-4 cm distal to the fracture site. A track is made with artery forceps up to the bone. A 5 mm drill sleeve (preferably with serrated end) is inserted up to the bone (Figure 1(e)). A 3.2 mm drill bit is used to drill the near cortex of the bone in the distal fragment. A 4.5 mm Schanz screw is inserted through the drill sleeve into the near cortex of distal fragment (Figure 1(f)). Now the surgeon controls the distal fragment with the Schanz screw and the proximal fragment with the help of cannulated reamer to achieve fracture reduction (Figure 1(g)), and the assistant inserts the guide wire through the cannulated reamer into the canal of distal fragment (Figure 1(h)). The Schanz screw is inserted in the intermediate fragment in segmental diaphyseal fractures (Type C2 fractures) (Figure 1(i)).

**Group II**

Fracture is reduced with traction through skeletal pin. The distal fragment is aligned with the proximal fragment primarily with traction. The guide wire is inserted into the canal of distal fragment after fracture reduction under C-arm.

### 2.3. Insertion of the Nail and Locking (Similar in Both Groups)

The intramedullary position of the guide wire in the distal fragment is confirmed under image intensifier. The reaming of the canal can be performed with stiff handheld cannulated reamers or flexible reamers up to the desired level. The smooth wire can be exchanged for a ball-tipped wire using the exchange sleeve if flexible reamers are used for reaming. An interlocking nail of appropriate size is inserted. Distal locking of the nail is performed with free hand technique in both groups. Proximal locking is performed using proximal interlocking guide. The following parameters were recorded for each procedure.

1. The guide wire insertion time: this was defined as the period beginning with the skin incision and ending with confirmation of intramedullary position of the guide wire in the distal fragment.
2. The radiation exposure during guide wire insertion: this was defined as the number of images required till confirmation of intramedullary position of the guide wire in the distal fragment.
3. Time for nail insertion and its distal locking: this was defined as the period between successful confirmation of intramedullary position of the guide wire in the distal fragment and the confirmation of accurate insertion of both distal screws.
4. The radiation exposure during nail insertion and its distal locking: this was defined as the number of images required for nail insertion and its distal locking.
5. Total operative time: this was defined as the period beginning with skin incision and ending with completion of skin closure.
6. The total radiation exposure: this was defined as the total number of images required during the operation.
7. The total fluoroscopic time: this was defined as the total radiation exposure time during the complete procedure as depicted on image intensifier screen.
8. The success or failure of the technique: a failure of the each technique was recorded when open reduction of fracture was necessary.

### 2.4. Statistical Analysis

Data were analyzed with Chi-square test with Yates' correction and student's *t*-test. For all tests, probability less than 0.05 was considered significant.

## 3. Results

Closed nailing was attempted in all cases and succeeded in 29 patients in group I and 25 patients in group II. Open reduction was required in one patient in group I (type C2 fracture) and five patients in group II (one type A fracture, two type B and two type C2 fractures). The use of cannulated reamer in the proximal fragment alone achieved closed reduction in 12 patients in group I. Schanz screw was used in the distal fragment or intact intermediate fragment in 18 patients in group I. The mean radiation exposures during guide wire insertion were 12.33 (range, 7–25) in group I and 22.1 (range, 9–31) in group II. The different values of other parameters are depicted in Table 1. Two patients in each group needed dynamization of the fracture to achieve union. Nonunion developed in one patient in group I and two patients in group II. Open exchange nailing using a large diameter nail with bone grafting from ipsilateral iliac crest was required to achieve union in these patients. No patient had fracture or infection at interlocking screw and Schanz screw sites. No cases of deep infection, avascular necrosis of femoral head, iatrogenic neurovascular injury, and fibrosis or quadriceps contracture were observed. Limb length shortening (range, 1–2.5 cm) was detected in one patient in group I and in two patients in group II. Angular (>5 degrees) or rotatory malalignment (>15 degrees) was observed in one patient in group I and 4 patients in group II.

### 3.1. Comparison of Groups I and II

No significant difference could be detected between groups I and II with respect to gender (*P* = 1), type of fracture (*P* = .92), need for open reduction (*P* = .197), nonunion (*P* = 1), and limb malalignments (*P* = .35). An unpaired *t*-test did not reveal significant differences between groups I and II with respect to the patient's age (*P* = .39), the average time for nail insertion and its distal locking (*P* = .94), the average total operative time (*P* = .35), and the average number of images taken during nail insertion and its distal locking (*P* = .39) (Table 1). The guide wire insertion time was significantly less in group I in comparison to group II (*P* = .002). The average number of images taken to achieve fracture reduction and guide wire insertion was less in group I versus group II. This decrease in radiation by 44% is statistically extremely significant at *P* < .0001. The average total number of images required during the complete procedure and average total fluoroscopic time were less in group I versus group II. This decrease in radiation by 22% is statistically extremely significant at *P* < .0001.

## 4. Discussion

The femoral shaft fractures in adults are preferably treated with closed intramedullary nailing [1–4]. Closed reduction is a critical component of the procedure. At times, closed reduction can be difficult and technically challenging [2, 19, 20]. In complicated cases, it may require prolonged exposure to ionising radiation during fluoroscopy of the fracture site, and when the fracture parts cannot be controlled, undesirable opening of the fracture haematoma is inevitable [20]. Simultaneous use of cannulated reamer in proximal fragment as intramedullary joystick and Schanz screw in the distal fragment as percutaneous joystick to achieve closed reduction has never been reported earlier. The present study aims to assess the effect of use of these instruments on radiation exposures and fluoroscopic and operative time required to achieve the fracture reduction during locked intramedullary nailing of femoral shaft fractures.

Numerous techniques and devices have been proposed to aid closed reduction [2, 3, 5–23] (Table 2). We agree with Aiyer et al. that preoperative skeletal traction is a crucial step and is the key to closed reduction and nailing [7]. Fracture table generates longitudinal traction to achieve closed reduction and maintains the reduction during the operative fixation [21]. Shezar et al. reported use of a mounted external supporting device (fixed to the fracture table) that can be controlled in both anterior-posterior and lateral planes to eliminate the deforming forces of thigh muscles [20]. A reliable assistant is of utmost importance to provide longitudinal traction for closed reduction when fracture table is not used [9, 11, 16]. McFerran and Johnson reported the use of a femoral distractor to aid in obtaining and holding a reduction [12]. Farrar and Binns used a Steinman pin on a T-clamp inserted percutaneously onto bone to gain a temporary reduction during intramedullary nailing [8]. Shewring et al. reported the use of an F-clamp to facilitate reduction and reduce exposure of the operator to radiation [19]. Oberst et al. reported use of intramedullary bone endoscopy for intramedullary fracture reduction under visual control to reduce the intraoperative use of an image intensifier [15]. A small bend at the end of guide wire can be used to assist with passage of guide wire into the distal fragment [14]. A small diameter nail in the proximal fragment [3, 10, 14, 22] or 8 mm straight reamer into the proximal fragment [7] and a Schanz pin as percutaneous skeletal joystick in either of the fragments [2] have been reported to aid closed reduction of the diaphyseal fractures. Sadighi et al. reported that Schanz screws provided a very effective method for closed reduction of femoral shaft fractures [18]. Ball spike pusher can also be used to achieve closed reduction and is applied to the bone through stab incisions like Schanz pins, thus respecting the fracture biology [16]. Afsari et al. reported clamp-assisted reduction through a small lateral incision to achieve accurate reduction of the subtrochanteric fractures [6]. Computerized navigation has the potential for increasing precision in fracture reduction while minimizing fluoroscopic requirements [5]. 

Ionizing radiation has no safe threshold of exposure below which it ceases to have adverse effects [26]. Moreover, long-term effects of this radiation exposure are unknown [27]. Therefore, every effort must be made to keep radiation exposure to minimum [26]. Use of cannulated reamer in the proximal fragment and Schanz pin in the distal fragment achieved closed reduction of the fracture with 44% decreased exposure to radiation in group I as compared to the group II. This decrease is extremely significant statistically (*P* < .0001). Moreover, the technique consumes less time for insertion of the guide wire in the distal fragment as reflected by statistically significant difference of the average guide wire insertion time. The present technique does not prolong the total operative time as the average total operative time was comparable in both groups (statistically insignificant). The technique utilizes readily available instruments and is not technically demanding.

The use of cannulated reamer in the proximal fragment alone achieved closed reduction in 40% patients in group I in the present study. The addition of the Schanz screw in the distal fragment achieved closed reduction in 96.6% patients in group I in comparison to 82.3% patients in group II in the present study. We believe that simultaneous use of cannulated reamer in proximal fragment as intramedullary joystick and Schanz screw in the distal fragment as percutaneous joystick further facilitates closed reduction of the fracture and insertion of the guide wire. The unicortical nature of the Schanz pin allows for passage of the guide wire. Use of Schanz screw offers the advantage of maintaining a closed soft tissue sleeve around the fracture, can be used with or without a fracture table, and allows for excellent control of the fracture fragments, using equipment that is readily available [2]. We are of the opinion that intramedullry joystick in the proximal fragment (small diameter nail or reamer) should preferably be used in all patients and the surgeon should keep a low threshold for percutaneous Schanz screw in the distal fragment. Another use of the technique is avoidance of angular malalignment as only one patient in group I had angular malalignment (3.3%) in the present series. Russell et al. also reported a similar technique that uses a percutaneous cannulated channel reamer over a guide pin, termed as minimally invasive nail insertion technique (MINIT), to avoid malreduction in proximal shaft fractures and reported malalignment in 5.2% when the MINIT was used and in 26% of the fractures treated without the use of MINIT [17]. Schanz screw can bend during skeletal manipulation. The surgeon should check the bending of the screw during operation to avoid its breakage inside the bone. 

The limitations of the present study include small number of patients in the series and potential for user bias because the surgeon could not be blinded with respect to the method used for closed reduction. However, the present study assessed impact of these devices on reduction of radiation exposure and need for open reduction during femoral nailing.

## 5. Conclusion

The use of cannulated reamer in proximal fragment as intramedullary joystick and Schanz screw in the distal fragment as percutaneous joystick facilitates closed reduction of the fracture during closed intramedullary femoral nailing with statistically significant reduction in guide wire insertion time and radiation exposure.

## Figures and Tables

**Table 1:** Comparison of the two groups.

	Group I		Group II		*P* value
	Mean	Standard deviation	Mean	Standard deviation	
The guide wire insertion time (min) (range)	26.57 (17–33)	4.55	30.87 (23–44)	5.55	Very significant (.002)
Time for nail insertion and its distal locking (min) (range)	27.93 (20–40)	4.68	27.83 (21–45)	6.34	NS (.94)
Total operative time (min) (range)	68.03 (55–81)	7.13	69.93 (51–82)	8.68	NS (.35)
The radiation exposure during guide wire insertion (range)	12.33 (7–25)	4.25	22.1 (9–31)	5.24	Extremely significant (*P* < .0001)
The radiation exposure during nail insertion and its distal locking (range)	25.27 (16–37)	4.52	26.17 (19–37)	5.04	NS (.47)
Total exposures in procedure (range)	37.6 (27–51)	6.62	48.27 (29–60)	7.59	Extremely significant (*P* < .0001)
Total fluoroscopic time (minutes) (range)	0.19 (0.13–0.25)	0.032	0.24 (0.14–0.3)	0.039	Extremely significant (*P* < .0001)

NS: Not significant.

**Table 2:** Different technique and devices to assist closed reduction of the fracture during intramedullary nailing.

Sr. No.	Reduction technique	References	Remarks
1	Preoperative skeletal traction	[2, 18, 26]	Key to closed reduction in delayed nailing
2	Traction on fracture table	[17, 22, 23]	
3	F-clamp	[19]	Facilitates reduction and reduces exposure of the operator to radiation
4	External supporting device	[20]	Eliminate the deforming forces of thigh muscles and reduces the radiation exposure
5	Strategically placed bumps	[16]	
6	Manual traction	[2, 6, 9, 16, 22, 23]	Decreased operative time
7	Femoral distracter	[10]	Useful in nailing without fracture table
8	Steinman pin on a T-clamp inserted percutaneously	[4]	
9	Percutaneous Schanz screws	[5, 18]	
10	Ball spike pusher	[16]	
11	Clamp-assisted reduction	[1]	Advocated clamp-assisted reduction with judicious use of a cerclage cable
12	A small diameter nail in the proximal fragment	[7, 14, 23, 26]	Kuntscher technique
13	8 mm straight reamer into the proximal fragment	[2]	
14	Percutaneous cannulated channel reamer over a guide pin	[17]	Significantly decreases the occurrence of malalignment in proximal femoral shaft fractures
15	Small bend at the end of guide wire	[14]	Corrects translation of the distal fragment
16	Intramedullary bone endoscopy	[15]	
17	Computerized navigation	[25]	Increases precision in fracture reduction while minimizing fluoroscopic requirements
18	Simultaneous use of cannulated reamer in proximal fragment and Schanz screw in the distal fragment	Present study	Reduces time and radiation exposure for closed reduction
